# Potential risk factors for the presence of anti-*Toxoplasma gondii* antibodies in finishing pigs on conventional farms in the Netherlands

**DOI:** 10.1186/s40813-022-00272-z

**Published:** 2022-06-14

**Authors:** Dorien M. Eppink, Martijn Bouwknegt, Joke W. B. van der Giessen, Manon Swanenburg, Derk Oorburg, Bert A. P. Urlings, Coen P. A. van Wagenberg, Marcel A. P. M. van Asseldonk, Henk J. Wisselink

**Affiliations:** 1 Vion Food Group, Boxtel, The Netherlands; 2 National Institute for Public Health and the Environment, Bilthoven, The Netherlands; 3 Wageningen Bioveterinary Research, Lelystad, The Netherlands; 4 Wageningen Economic Research, Wageningen, The Netherlands; 5 Van Hall Larenstein, Velp, The Netherlands

**Keywords:** *Toxoplasma gondii*, Pigs, Risk factors, Seroprevalence, Cats

## Abstract

**Background:**

The parasite *Toxoplasma gondii (T. gondii)* causes a substantial human disease burden worldwide. Ingesting improperly cooked pork containing *T. gondii* is considered one of the major sources of human infection in Europe and North America. Consequently, control of *T. gondii* infections in pigs is warranted. The European Food Safety Authority advised to perform serological monitoring of pigs and to conduct farm audits for the presence of risk factors. Serological monitoring was implemented in several Dutch slaughterhouses, one to six blood samples (a total of 5134 samples) were taken from each delivery of finishing pigs and samples were tested for the presence of anti-*T. gondii* antibodies. Using these test results, a cross-sectional study was initiated to assess the association between the within-herd *T. gondii* seroprevalence and the presence of risk factors for *T. gondii* infections at 69 conventional finishing pig farms in the Netherlands.

**Results:**

A multivariable model showed significant (*P* ≤ 0.05) association with twelve potential risk factors: type of farm, presence of dogs, presence of ruminants, use of boots, use of shower and farm clothing, mode of rodent control, bedding accessibility for rodents, presence of cats, type of drinking water, heating of the feed, use of goat whey and shielding of birds.

**Conclusions:**

Serological monitoring of finishing pigs for *T. gondii* in slaughterhouses can be used to identify the presence of *T. gondii* risk factors on Dutch conventional finishing pig farms and seems a valuable tool to guide and monitor the control of *T. gondii* in pork production.

**Supplementary Information:**

The online version contains supplementary material available at 10.1186/s40813-022-00272-z.

## Background

Globally, *Toxoplasma gondii* (*T. gondii*) is recognized as a pathogen causing a substantial human disease burden [1]. It is estimated that up to one third of the world population has been exposed to the parasite [2]. In the Netherlands, toxoplasmosis ranks second on a list of prioritized emerging zoonoses [3] and also second in disease burden among 14 food-related pathogens [4].

*T. gondii* is an intracellular protozoan zoonotic parasite. Although sexual reproduction is only possible in felids, the definitive host, it can probably infect almost all warm-blooded animals including humans [5]. Human infection with *T. gondii* can occur by ingestion of sporulated oocysts present in soil or water, by ingestion of contaminated fruit or vegetables or raw or undercooked meat from infected animals [5]. In humans, vertical transmission may occur from mother to unborn child. Finally, transmission may occur via blood transfusion or organ transplantation [6].

Ingesting raw or undercooked meat is one of the major sources of human *T. gondii* infection in Europe and North America [7–9]. In the Netherlands, pork contributed approximately 12% to the total meat-borne *T. gondii* infections [10]. Consequently, because of the high human disease burden of *T. gondii*, control of *T. gondii* infections in pigs is warranted.

Pigs can be infected in two ways, either by ingestion of *T. gondii* sporulated oocysts in contaminated feed or water or by ingestion of bradyzoites infected rodents or birds. Few pigs become infected prenatally by trans-placental transmission [1]. Although the parasite can cause illness and mortality, especially in neonatal pigs, most pigs show few clinical signs [1, 11, 12]. The level of *T. gondii* infections in pig herds depends on the farming system; outdoor access leads to a higher reported seroprevalence compared to being held solely indoors [13–15]. Other reported risk factors for *T. gondii* infection include the presence of cats, rodents and flies on the farm, the accessibility of cats, rodents and birds to pig feed, water and enrichment material, the feeding of goat whey and the degree of cleaning and disinfection on the farm [1, 2, 14, 16–23].

By the currently practiced meat inspections at slaughter detection of *T. gondii* in carcasses is impossible due to the small size of tissue cysts and absence of pathological changes in carcasses [13]. To control *T. gondii* infections in pigs, the European Food Safety Authority (EFSA) advised to perform serological monitoring of pigs and to conduct farm audits for the presence of risk factors [13]. Indirect (serological) methods, based on the detection of antibodies against the parasite, have been developed [1, 24]. Among these methods, enzyme-linked immunosorbent-assay (ELISA) techniques have been used and validated for the diagnosis of *T. gondii* infection in pigs. These assays are easy to perform and enable testing of large numbers of serum samples within a short time. In addition, several ELISA tests have been standardized and commercialized [24–26].

Serological monitoring for *T. gondii* was implemented in several Dutch slaughterhouses [27]. Results from Swanenburg et al. (2019) showed that seroprevalence varied over years, from 1.4 to 2.8% during a five year study period from 2012 to 2016 [27]. Samples from all batches of finishing pigs were tested for the presence of anti-*T. gondii* antibodies. Based on these results, a cross-sectional study was initiated to assess the association between the within-herd *T. gondii* seroprevalence and the presence of risk factors for *T. gondii* infections at finishing pig farms in the Netherlands.

## Results

### Descriptive results

A total of 69 farmers agreed to participate in this study. Approximately 5% of the initially contacted farmers declined to cooperate. Reasons for declining participating were: farm biosecurity, lack of time, lack of motivation or a (temporary) cessation of raising pigs. Fourteen of the participating farms were farrow-to-finish operations. Of the 5134 serum samples tested from the participating farms 5% (259) were considered positive for anti-*T. gondii* antibodies. Twenty-five farms had no positive blood samples (Table 1).

**Table 1:** Frequency distribution (*n*) of 69 Dutch finishing pig farms, their tested sera, and the positive tested sera related to the percentage of within-farm *T. gondii* positive seroprevalence

Seroprevalence at finishing pig farm (%)	*N* (%) farms	*N* sera tested	*N* sera positive
0	25 (36)	800	0
0–5	19 (27)	2836	51
5–10	8 (12)	555	33
10–20	11 (16)	744	106
> 20	6 (9)	199	69
Total	69 (100)	5134	259

### Farm and management characteristics related to the within-farm T. gondii seroprevalence

In total, 25 of the 30 examined potential risk factors reached a *P* ≤ 0.15 in univariable logistic regression (Table 2). One risk factor (‘feeding compost, soil or peat’) had too few observations in a category to have the model run adequately and was excluded from the multivariable analysis. The risk factor ‘use of straw’ was excluded from the multivariable analysis due to missing values. The variables ‘wet/liquid feed’, ‘roughage’ and ‘corncob mix’ were excluded from the multivariable analysis due to correlations with the variables ‘compound feed heated’ (i.e. correlation coefficient [r] > 0.7), ‘bedding pigs accessible for rodents’ (i.e. r > 0.7) and ‘pig feed accessible for cats’ (i.e. r > 0.5), respectively. The variables ‘pig feed accessible for rodents’ and ‘pig feed accessible for cats’ were strongly correlated (i.e. r > 0.7). Given cats are the definitive host, the variable ‘pig feed accessible for cats’ was retained. Correlations between other variables were ≤ 0.5.

**Table 2:** Univariate analysis of potential risk factors for *T. gondii* infection in pigs from 69 Dutch finishing pig farms

Risk factor	Categories	No. of Farms	Blood samples			*P*-value
			No. positive	No. tested	Avg. % pos.*	
Type of farm	Closed	14	42	1180	6.1%	0.008
	Open	55	217	3954	7.0%	
Presence of dogs	Absent	22	83	1211	9.6%	0.002
	Present	47	176	3923	5.5%	
Presence of poultry	Absent	59	170	4276	5.1%	< 0.0001
	Present	10	89	858	16.7%	
Presence of ruminants^†^	Absent	41	145	3463	5.2%	< 0.0001
	Present	28	114	1671	9.1%	
Well-defined clean/dirty zones	No	52	217	3802	7.1%	0.000
	Yes	17	42	1332	5.9%	
Boots only used inside stables	No	43	207	3578	8.6%	0.000
	Yes	26	52	1556	3.9%	
Shower and farm clothing	No	61	235	3947	7.2%	< 0.0001
	Yes	8	24	1187	4.0%	
Purchase of breeding gilts^a^	No	58	228	4376	6.8%	0.195
	Yes	11	31	758	6.9%	
Cleaning every round of pigs	No	21	115	1472	6.3%	< 0.0001
	Yes	47	136	3244	7.1%	
Presence of cats	No	19	47	1530	5.1%	< 0.0001
	Yes, no stable access, no kittens spotted	29	82	2498	4.1%	
	Yes, no stable access, kittens spotted	11	82	697	9.9%	
	Yes, with stable access, no kittens spotted	3	7	99	5.7%	
	Yes, with stable access, kittens spotted	6	41	304	21.3%	
Pig feed accessible for cats	No	46	83	2972	2.7%	
	Yes	23	176	2162	15.0%	
Bedding pigs accessible for cats^a^	No	65	253	5008	6.8%	0.882
	Yes	4	6	126	6.4%	
Feed heated	No	37	195	3459	9.0%	0.004
	Yes	32	64	1675	4.2%	
Compost, soil, peat^b^	No	68	249	5038	6.7%	0.032
	Yes	1	10	96	10.4%	
Whey (goat and/or cow)	No	51	134	3012	5.1%	0.021
	Yes	18	125	2122	11.5%	
Whey (cow)^a^	No	59	220	4226	6.3%	0.246
	Yes	10	39	908	9.8%	
Whey (goat)	No	65	201	4887	4.9%	< 0.0001
	Yes	4	58	247	37.6%	
Wet/Liquid feed^c^	No	35	75	1702	4.7%	0.137
	Yes	34	184	3432	8.9%	
Roughage^c,d^	No	61	252	4352	7.3%	< 0.0001
	Yes	6	6	478	4.3%	
Corncob mix^c,d^	No	52	132	3001	5.2%	0.000
	Yes	15	126	1829	13.3%	
Use of straw^d^	No	54	227	4054	7.5%	0.059
	Yes	13	31	776	5.0%	
Garden/kitchen waste	No	66	244	5030	6.6%	0.000
	Yes	3	15	104	11.5%	
Pig drinking water	Tapwater	32	96	1555	7.0%	0.017
	Well	37	163	3579	6.6%	
Shielding of flies	No	49	148	3349	6.1%	0.006
	Yes	20	111	1785	8.5%	
Shielding of birds	No	8	26	316	9.1%	0.014
	Yes	61	233	4818	6.5%	
Professional rodent control^a^	No	41	145	2652	7.2%	0.152
	Yes	28	114	2482	6.2%	
Mode of rodent control	No or trap-only control	4	12	262	4.0%	0.051
	Poisson-control	47	175	3808	6.4%	
	Poisson and trap-control	18	72	1064	8.5%	
Stable accessible for rodents^a^	No	31	113	2188	4.6%	0.736
	Yes	38	146	2946	8.6%	
Pig feed accessible for rodents^c^	No	36	51	1877	2.3%	< 0.0001
	Yes	33	208	3257	11.7%	
Bedding pigs accessible for rodents	No	64	253	4767	6.9%	0.004
	Yes	5	6	367	5.1%	

*Average of the % positive samples at farm level

^†^Cattle, sheep and/or goats

^a^Risk factors not included in the multivariable analysis due to *P* > 0*.*15 in univariable analysis

^b^Risk factors not included in the multivariable analysis due to low frequency counts

^c^Risk factors not included in the multivariable analysis due to collinearity issues

^d^Risk factors not included in the multivariable analysis due to missing values

In the most parsimonious multivariable model 12 variables were significantly (*P* ≤ 0.05) associated with the presence of antibodies positive blood serum samples for *T. gondii* on 69 farms (Table 3).

**Table 3:** Multivariable analysis of potential risk factors for *T. gondii* infection in finishing pigs from 69 Dutch finishing pig farms using backward elimination and inclusion criterion of *P* ≤ 0.05

Risk factor	No. of Farms	Blood samples			OR	95% CI	*P*-value
Variables		No. positive	No. tested	Avg. % pos.*			
*Type of farm*							
Closed	14	42	1180	6.1%	1.00		0.0488
Open	55	217	3954	7.2%	0.63	0.40–1.00	
*Dogs*							
Absent	22	83	1211	9.6%	1.00		0.0161
Present	47	176	3923	5.7%	0.60	0.40–0.91	
*Ruminants (cattle, sheep and/or goat)*							
Absent	41	145	3463	5.4%	1.00		0.0071
Present	28	114	1671	9.1%	1.67	1.15–2.42	
*Boots only used inside stables*							
No	43	207	3578	8.7%	1.00		0.0068
Yes	26	52	1556	4.0%	1.91	1.20–3.04	
*Shower and farm clothing*							
No	61	235	3947	7.3%	1.00		0.0106
Yes	8	24	1187	4.3%	0.37	0.17–0.79	
*Mode of rodent control*							
No or only with traps	4	12	262	4.0%	1.00		0.0056
Poison	47	175	3808	6.5%	3.37	1.23–9.23	
Poison and traps	18	72	1064	14.1%	5.57	1.90–16.3	
*Bedding pigs accessible for rodents*							
No	64	253	4767	7.1%	1.00		0.0002
Yes	5	6	367	5.1%	0.17	0.07–0.44	
*Presence of cats*							
Absent	19	47	1530	5.1%	1.00		< 0.0001
Present, no kittens, no stable access	29	82	2498	4.2%	1.90	1.11–3.27	
Present, kittens, cats not in stable	11	82	697	9.9%	11.80	6.23–22.5	
Present, no kittens, stable accessible	3	7	99	5.7%	2.87	0.67–12.3	
Present, kittens, stable accessible	6	41	304	21.3%	4.20	2.04–8.55	
*Drinking water for pigs*							
Tapwater	32	96	1555	7.3%	1.00		0.0095
Well	37	163	3579	6.7%	0.60	0.40–0.88	
*Feed heated*							
No	37	195	3459	9.2%	1.00		0.0129
Yes	32	64	1675	4.3%	0.42	0.21–0.83	
*Whey (goat)*							
No	65	201	4887	5.0%	1.00		< 0.0001
Yes	4	58	247	37.6%	11.30	7.12–18.0	
*Shielding of birds*							
No	8	26	316	9.1%	1.00		0.0035
Yes	61	233	4818	6.7%	0.18	0.06–0.57	

*Average of the % positive samples at farm level

## Discussion

In this study the association between *T. gondii* seroprevalence and potential risk factors for *T. gondii* infections in finishing pig herds in the Netherlands was assessed. Twelve out of 30 variables were identified as potential risk factors. Most of these 12 potential risk factors are already well known for *T. gondii* and in general related to the presence of cats, presence of other animals, the accessibility of cats, rodents and birds to the stables and feeds and mode of rodent control [18]. To determine the association between seroprevalence and risk factors, the seroprevalence in the selected herds was taken from a serological surveillance system at the slaughterhouses; from every delivery of finishing pigs to the slaughterhouse one to six serum samples were taken and tested for anti *T. gondii* antibodies [27]. Therefore, we can conclude that this serological surveillance system can be used to identify finishing pig farms where the typical *T. gondii* risk factors are present and monitor the control of *T. gondii* in pig herds. Recently, we performed an intervention study on five pig farms in which the within-herd *T. gondii* seroprevalence was successfully used to evaluate the effectiveness of the interventions on *T. gondii* risk factors [28]. These results confirm that determination of within-herd *T. gondii* seroprevalence is a useful part of a surveillance system based on serology for detection of *T. gondii* infections in pigs.

As in other studies, in our study the presence of cats at the barnyard or in the pig stables was associated with increased anti-*T. gondii* seroprevalence in pigs. Pigs can get infected with *T. gondii* by uptake of soil, feed and water contaminated with oocysts that were shed by cats, or by ingestion of cysts in the tissues of infected intermediate hosts, for example rodents and birds [29].

Our results also showed that not just the presence of cats on pig farms is a significant risk factor but that this significance increased when kittens were present. Kittens pose the highest risk of spreading oocysts in the environment, because most cats are infected with *T. gondii* as juveniles [30] or even as suckling kittens [31]. Cats only spread *T. gondii* in their feces for 1–3 weeks following the first episode of infection and they become immune to re-shedding of oocysts [32]. Neutering adult cats to prevent kittens to be born was found to be a successful intervention to achieve a significant reduction in *T. gondii* seroprevalence in a pig herd [28]. On farms, cats are often used to control rats and mice. Rats and mice are also a risk factor for *T. gondii* infection and for reduced biosecurity. Thus, many pig farmers might not want to remove all cats from the farm. As an alternative to the advice of removing all cats from the farm, it can be advised to neuter cats to prevent kittens on the farm.

Our questionnaire included several questions about feed-related variables, because uptake by pigs of sporulated oocysts of *T. gondii* in animal feed is an important route by which pigs can be infected. Open or less confined feed storage or feeding area represent an increased risk for exposure of livestock to the parasite [29]. However, most of the feed-related variables could not be analyzed in our multivariable analysis due to collinearity with other variables or due to missing values. The only feed-related variable included in the multivariable analysis was the use of heated feed, and this was associated with lower *T. gondii* seroprevalence. High temperatures during the production of pig feed can inactivate the parasite. More research is needed to analyze the impact of other feed-related variables.

Feeding goat whey was associated with a higher seroprevalence. Although there were only four farms that fed goat whey, the difference in seroprevalence with the other 63 farms was considerable (OR of 11.30). Our observations are in line with other studies that showed that feeding unheated goat whey to pigs is an important risk factor for infection with *T. gondii* [20, 31].

As in other studies, the mode of rodent control was identified as a risk factor for *T. gondii* infections in pigs [14, 17]. Besides that, in this study we found that the mode of rodent control mattered; control with a combination of poison and traps has a higher OR than the use of poison and traps separately. This suggests that simultaneous application of the two approaches for rodent control could be more effective than a single approach alone.

As in other studies, we identified shielding of birds as a preventive factor for *T. gondii* infections in pig herds [29]. Birds can acquire *T. gondii* infection through ingestion of oocysts from the ground or through ingestion of tissue cysts present in infected prey. Like rodents, birds are incidentally caught and eaten by pigs.

In our study, presence of other farm animals (cattle, sheep and/or goats) on the farm was found to be a risk factor for *T. gondii* infection in finishing pigs, while in other studies it was not [19, 33]. However, in line with our findings, a recent review [29] suggested that the presence of multiple animal species on a farm could serve as an indicator of low farming intensity and that this low intensity was often related to a higher risk of *T. gondii* seropositivity.

In our study, presence of dogs on the farm was found to be a preventive factor for *T. gondii* infection in pigs (Odds Ration [OR] = 0.5). Hill et al. (2010) also observed that the presence of dogs reduced the number of *T. gondii* seropositive samples on surveyed farms, indicating that dogs could control rodents [16]. Furthermore, dogs could also deter cats and thereby deterring the main reservoir of *T. gondii*. However, other studies identified the presence of dogs as a significant risk factor for *T. gondii* infection in pigs [14, 34] or did not find a significant association [23].

The use of boots only in the stables was identified as potential risk factor, although the crude percentage of positive samples was lower for this category compared to the reference. A similar apparent mismatch was observed for the variable ‘type of farms’. Additional modelling (forward multivariable selection, interaction terms, bi- and trivariable logistic regression; data not included), showed that confounding and effect modification/interaction were unlikely to explain this observation. We hypothesize that these observations result from the effect explained by other variables in the multivariable model. The remaining effect attributed to the two mentioned risk factors is thus opposite to what one would initially expect.

In our study, use of well water was found as a preventive factor for *T. gondii* infection compared to tap water. A recent review [30] concluded that it is hard to quantify the risk for a *T. gondii* infection of pigs through well water, because in some studies well water was associated with an increased risk, while it seemed to have a protective statistical effect in others. A potential reason for these differences could be that in some studies cats had access to the water before it reached the pigs and contaminated it with oocysts, whereas in other studies they did not. Water can be supplied to the pigs from a variety of sources and in different ways, depending on the production system and regional circumstances. It might not be possible for a pig farmer to change the water source to control *T. gondii* infection, because the production system prescribes a specific water source or regional circumstances prevent implementation of certain water sources.

## Conclusion

Twelve potential risk factors for *T. gondii* infection of finishing pigs were identified using serological screening of Dutch conventional pig farms. The use of serological surveillance seems therefore a valuable tool to guide and monitor the control of *T. gondii* in pork production.

## Materials and methods

### Farm selection and study

Multiple Dutch slaughterhouses owned by one company ran a serological monitoring program for *T. gondii*. From every delivery of finishing pigs, a minimum of one and a maximum of six serum samples were taken [27]. The serum samples were tested for anti-*T. gondii* antibodies with a PrioCHECK™ Toxoplasma Antibody ELISA (Thermo Fisher Scientific Prionics B.V., Lelystad The Netherlands) [24, 26]. A sample was classified as positive if it exceeded 20% positivity (PP), as described by the manufacturer. Initially this study started in 2015 as a case–control study. The selection criteria for case farms were: active supplier (minimal 6 deliveries per year), *T. gondii* prevalence in slaughter pigs of minimal 15% in the previous year (with a test cut-off of PP20) and minimally one serologically positive *T. gondii* result in the last 8 months. The selection criteria for control farms were: active supplier (minimal 5 deliveries per year), delivers approximately the same number of pigs to the slaughterhouse as the matched case farm and negative Toxoplasmosis results in the last 12 months. After re-evaluation of preliminary results it was found that the classification of the control farms was incorrect, as it turned out that some of the farms appeared to have positive samples after all. Therefore, we approached the study as a cross-sectional study, realizing that between-farm seroprevalence estimates will be biased from the true between-farm seroprevalence due to the selection procedure. Estimating the between-farm seroprevalence was, however, not our objective and thus not considered problematic. The within-herd seroprevalence was estimated for each farm using the test results from the 12 months preceding questionnaire completion. The total study period was from 2015 to 2019.

### Questionnaire

To identify the most important control measures to prevent, reduce or control the introduction and spread of *T. gondii* on a pig farm, participating farmers were audited using a questionnaire. Our questionnaire was based on an earlier developed questionnaire [18] and used the Hazard Analysis Critical Control Points (HACCP) framework. The questionnaire contained questions about farm and management characteristics potentially related to *T. gondii* infection in the pigs, general farm biosecurity measures, outdoor access, rodent control, presence of cats, type of feed and water supply (Table 1, Additional file 1). The full questionnaire is available as Additional file 1 and the detailed results per farm as Additional file 2. During farm visits, a project researcher completed the questionnaire by interviewing the farmer. Furthermore, the interior and the outside environment of the stables were subjected to a visual inspection to verify elicited answers.

### Statistical analysis

The effect of possible risk factors on the within-herd seroprevalence of anti-*T. gondii* antibodies was assessed using logistic regression [35]. The presence of antibodies in a blood serum sample was considered a binomial process, with the number of blood serum samples taken from farm *i*, *n*_*i*_, being the number of trials. The probability of a test-positive sample (i.e., the within-farm seroprevalence) for farm *i* was the dependent variable and expressed as number of positive samples (*k*_*i*_) over *n*_*i*_.

The statistical analysis was done in SAS program version 9.4 (SAS Institute, Cary, NC, USA). Univariable analysis was used to preselect variables for multivariable analysis, where *RF*_*j*_ showing a probability < 0.15 were selected. Correlation between selected variables was assessed via the Pearson’s correlation coefficient. If that coefficient was >|0.5|, then the correlated RF with the most likely biological explanation was included. The multivariable model was trimmed through a backward procedure as described by Hosmer and Lemeshow [35] and was considered completed when remaining variables all had a *P*-value < 0.05. Two-way interaction terms were added one by one to check for statistically significant interaction terms (*P*-value < 0.05). The fit of the multivariable model was assessed with Hosmer and Lemeshow goodness-of-fit test.

## Supplementary Information

**Additional file 1. **HACCP-based questionnaire.

**Additional file 2.** Toxoplasma data of questionnaire.

## Data Availability

All data generated or analysed during this study are included in this published article [and its Additional files].
