# Supplementary material for: Potential risk factors for the presence of anti-Toxoplasma gondii antibodies in finishing pigs on conventional farms in the Netherlands
Source: Porcine Health Manag. 2022 Jun 14;8:27. doi: 10.1186/s40813-022-00272-z (PMC9195196; doi:10.1186/s40813-022-00272-z)
Supplement: Supplementary file 1 — Additional file 1. HACCP-based questionnaire. [file 40813_2022_272_MOESM1_ESM.docx]

| Project researcher: ........…………………………………………………………………………………………….  Date of visit: ……………………………………………………….  Name of pig farmer / company name: ……………………………………………………………………..  Place: …………………………………………………………………....  UBN: ………………………………………………………………………………………………………………........….  Contact details farmer: …………………………………………………………………................... |
| --- |

| **General farm characteristics** |
| --- |

1. Type of farm:

- Finishers
- Closed farm
- Partly closed ……%

1. Number of finishers: ……………………………………………
2. Number of sows:……………………………………………………
3. Do you own other locations where you keep pigs? ………….. locations
4. Distance to nearest village?

- < 1km
- > 1 km

1. Do you perform other business activities at this location which attribute to your income?

- Yes, these are…………………………………………………………………………………
- No

1. Are there other animals present on this farm location besides pigs?

- Yes
- No

7a. If yes, which animals are present on this farm location?

- Cattle
- Sheep
- Goats
- Cats
- Dogs
- Poultry
- Other, which are ..........................

| **Supply of pigs** |
| --- |

1. Do you purchase breeding gilts?

- Yes
- No

| **Outdoor access** |
| --- |

1. Do the finishers have access to an outdoor area?

- Yes
- No

9a. If yes, what is the substrate of the outdoor area?

- Concrete
- Soil
- Other: ……………………………………….

1. Do the sows have access to an outdoor area?

- Yes
- No
- Only a percentage of the sows, …..

10a. If yes, what is the substrate of the outdoor area?

- Concrete
- Soil
- Other: …………………………..

| **Biosecurity** |
| --- |

1. Is there a well-defined separation between the clean and dirty zones?

- Yes
- No, but the farmer makes a distinction between the zones
- No, because there is no separation possible

1. Do other animals than pigs, have access to the clean zone?

- Yes
- No

1. What is the state of the biosecurity corridor?

- There is no biosecurity corridor
- There are only boots available for visitors
- There are boots and an overall available for visitors
- Visitors need to shower before entering
- Other: ......................................................

1. Does every stable have their own boots (when access is via outdoor)?

- Yes
- No

1. Are the pens of the pigs cleaned in between every round of pigs?

- Yes
- No

| **Pest control and prevention** |
| --- |

1. Do you perform control of flies?

- Yes, how? …………………………………….
- No

1. Do you shield of flies from outside?

- Yes, how? …………………………………..
- No

1. Do you shield of birds?

- Yes, how? …………………………………
- No

1. How do you rate the accessibility of rodents to the stable?

- Easy
- Hard

1. Do rodents have access to the feed or the feed storage?

- Yes
- No, because ....................

1. Do rodents have access to the enrichment material or bedding?

- Yes
- No, because ..................................

1. Do you perform rodent control?

- Yes, very consistent
- Yes, though I could spend more time on it
- No

22a. If yes, do you perform rodent control yourself or by an employee or via a professional rodent control company?

- Farmer or employee
- Professional rodent control

22b. How is the rodent control performed?

- Use of clips/traps
- Use of poison
- Other: ………………………………………………

| **Presence of cats** |
| --- |

1. Are there cats present at the farm?

- Yes, 1 – 3 cats
- Yes, > 3 cats
- No

1. What is the age of the cats present?

- < 2 years
- > 2 years
- Both

1. Were there kittens born last year?

- Yes
- No

1. Do you see, on a regular base, other cats then you own on the farm?

- Yes
- No

1. Do cats have access to the outdoor area of the pigs?

- Yes
- No

1. Do cats have access to the stable?

- Yes
- No

1. Do cats have access to the feed or feed storage?

- Yes
- No, because .............................

1. Do cats have access to the enrichment material or bedding?

- Yes
- No, because ..................................

| **Water supply** |
| --- |

1. What is the source of your water supply to the pigs?

- Mains
- Well
- Rain water
- Other

1. Do the pigs have access to outside water?

- Yes
- No

1. How is the water supplied to your pigs?

- Via float bins
- Via pipes
- Other: ................................................

| **Feed supply** |
| --- |

1. Do the pigs only get compound feed which is heated above 65 degrees Celsius?

- Yes
- No

1. Do the pigs get additional compost, soil or peat fed?

- Yes
- No

1. Do you feed raw milk goat whey to your pigs?

- Yes
- No

1. Do you feed raw milk cow whey to your pigs?

- Yes
- No

| 1. Part of the feeding/management program? | Yes/No |
| --- | --- |
| Roughage |  |
| Hay |  |
| Wet/liquid feed |  |
| Loose straw |  |
| Bedding material |  |
| Garden/kitchen waste |  |

| **Appearance of the farm** |
| --- |

1. What is the appearance of the farm from the outside?

- Very messy
- Messy
- Tidy
- Very tidy
